# Recent advances in bioluminescent probes for neurobiology

**DOI:** 10.1117/1.NPh.11.2.024204

**Published:** 2024-02-22

**Authors:** Katherine M. Townsend, Jennifer A. Prescher

**Affiliations:** a University of California, Irvine, Department of Chemistry, Irvine, California, United States; b University of California, Irvine, Department of Molecular Biology and Biochemistry, Irvine, California, United States; c University of California, Irvine, Department of Pharmaceutical Sciences, Irvine, California, United States

**Keywords:** bioluminescence, luciferin, luciferase, neurobiology, imaging

## Abstract

Bioluminescence is a popular modality for imaging in living organisms. The platform relies on enzymatically (luciferase) generated light via the oxidation of small molecule luciferins. Since no external light is needed for photon production, there are no concerns with background autofluorescence or photobleaching over time—features that have historically limited other optical readouts. Bioluminescence is thus routinely used for longitudinal tracking across whole animals. Applications in the brain, though, have been more challenging due to a lack of sufficiently bioavailable, bright, and easily multiplexed probes. Recent years have seen the development of designer luciferase and luciferin pairs that address these issues, providing more sensitive and real-time readouts of biochemical features relevant to neurobiology. This review highlights many of the advances in bioluminescent probe design, with a focus on the small molecule light emitter, the luciferin. Specific efforts to improve luciferin pharmacokinetics and tissue-penetrant emission are covered, in addition to applications that such probes have enabled. The continued development of improved bioluminescent probes will aid in illuminating critical neurochemical processes in the brain.

## 1 Introduction

Bioluminescence is a powerful technology for visualizing biological processes in live animals.^1^^,^^2^ This platform relies on luciferase enzymes that oxidize small molecule luciferins to produce light.^3^ Photons that escape the subject are then registered by external cameras to generate images. Since light production requires no external excitation source, little-to-no background signal is produced and issues associated with phototoxicity or bleaching are avoided. Bioluminescence is thus an attractive choice for serial imaging studies in whole animals. Indeed, luciferases and luciferins have long been used for noninvasive tracking of cell proliferation and gene expression (especially in the periphery), due to widely accessible reporter lines and bioavailable substrates.^3^^–^^7^

**Fig. 1:**
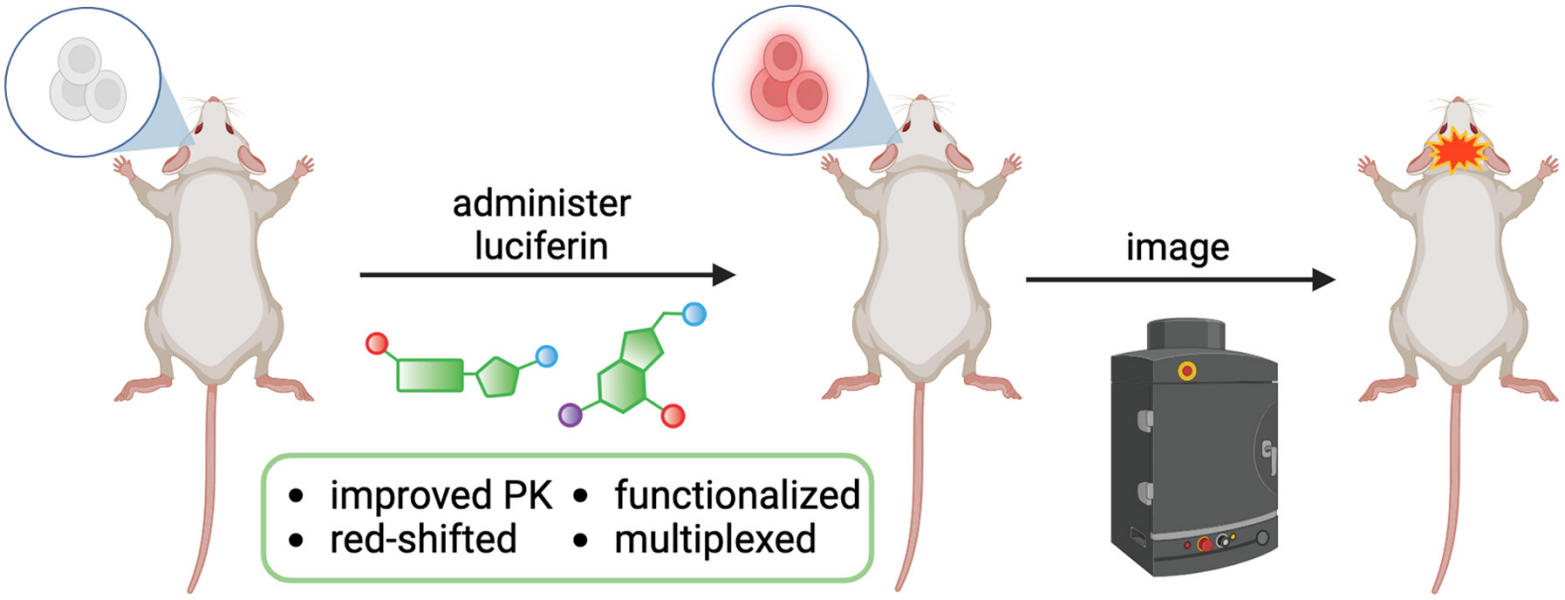
General workflow for BLI in the brain. Luciferase-expressing cells can be illuminated upon luciferin administration. Images are typically acquired using a cooled CCD camera, with subjects placed in a light-tight chamber. Advances in luciferin design discussed in this review are highlighted.

Applications in the brain have been somewhat more challenging, given the historic difficulties of delivering probes to this region. Luciferase reporters are typically localized via direct cell implantation or viral gene delivery. Luciferin analogs are then administered to illuminate the target cells or molecular event (Fig. 1).^2^^,^^8^ Classic tools can suffer from diminished photon outputs or suboptimal pharmacokinetics in brain tissue. Many of the emitted photons also evade detection due to the highly scattering environment. It has thus been challenging to use bioluminescent probes for examining many aspects of neurobiology in live animals.

These limitations have spurred several efforts to improve the sensitivity of bioluminescence imaging (BLI) and expand its applications in neuroscience. While early efforts focused on optimizing the luciferase component, many recent advances have focused on modifying the small molecule luciferin. The end result has been a series of analogs with altered colors of emission, as well as enhanced blood-brain-barrier penetration and overall biodistribution.^9^ Water-soluble probes that emit bright, red-shifted light are desirable, to both maximize the amount of probe that can be delivered and the number of photons that penetrate surrounding tissue. Luciferins can further be outfitted with custom “cages”; removal of the cage can be tied to a number of bioactivities, providing a means to image (or sense) these processes.^10^^,^^11^ Continued work on designer luciferin-luciferase probe sets is also lowering the barrier to imaging discrete biomolecules involved in brain function.^10^

In this review, we summarize recent advances in luciferin design for improved BLI, with a focus on applications in neuroscience. We first focus on improvements in luciferin pharmacokinetics and tissue-penetrant emission. We then highlight advances in the development of functional luciferins—molecules that can be used for metabolite sensing, optogenetics, and other applications. Finally, multicomponent imaging applications and emerging classes of probes will be discussed.

## 2 Brief History

**Fig. 2:**
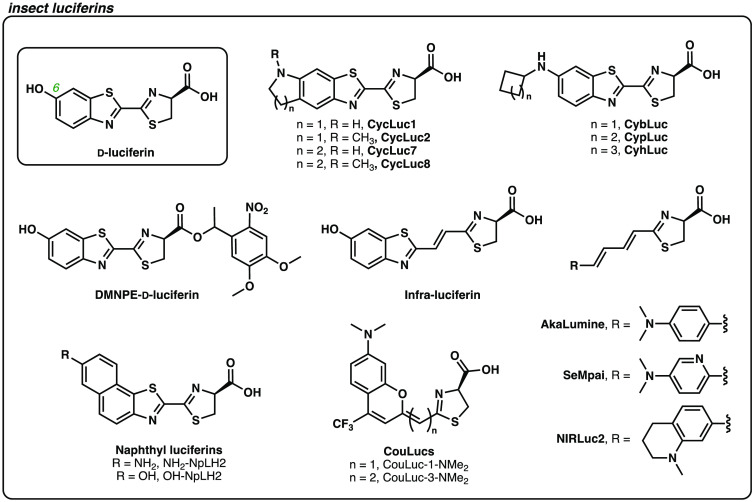
Luciferin analogs based on the insect substrate, D-luciferin.

To date, most BLI studies in mice have leveraged luciferases and luciferins from the insect family. Arguably the most popular set is firefly luciferase (Fluc) and its cognate substrate, D-luciferin (Fig. 2).^12^ Light production from this pair (and all insect variants) requires molecular oxygen and adenosine triphosphate (ATP). The light produced is mostly yellow-green in color, although enough tissue-penetrant photons (>650  nm) are generated for *in vivo* detection.^13^^,^^14^ Insect luciferases give off the largest percentage of red-light among common bioluminescent enzymes, making them attractive for use in mouse models. Red light is less absorbed and scattered by endogenous chromophores (i.e., hemoglobin and melanin) in tissue, resulting in less signal attenuation.^2^ D-Luciferin is also relatively stable and sufficiently bioavailable to peripheral tissues upon standard intraperitoneal injection.^15^ For these reasons, D-luciferin and Fluc are used ubiquitously for visualizing biological processes in live animals.^7^^,^^14^^,^^16^ Imaging in deep locales (like the brain), though, has been historically challenging.^1^^,^^17^ Some limitations result from poor pharmacokinetics of the small molecule substrates. Previous biodistribution studies with radiolabeled D-luciferin showed low accumulation in the brain.^18^ Additionally, Fluc and related insect luciferases exhibit quite slow turnover rates, limiting photon outputs.

**Fig. 3:**
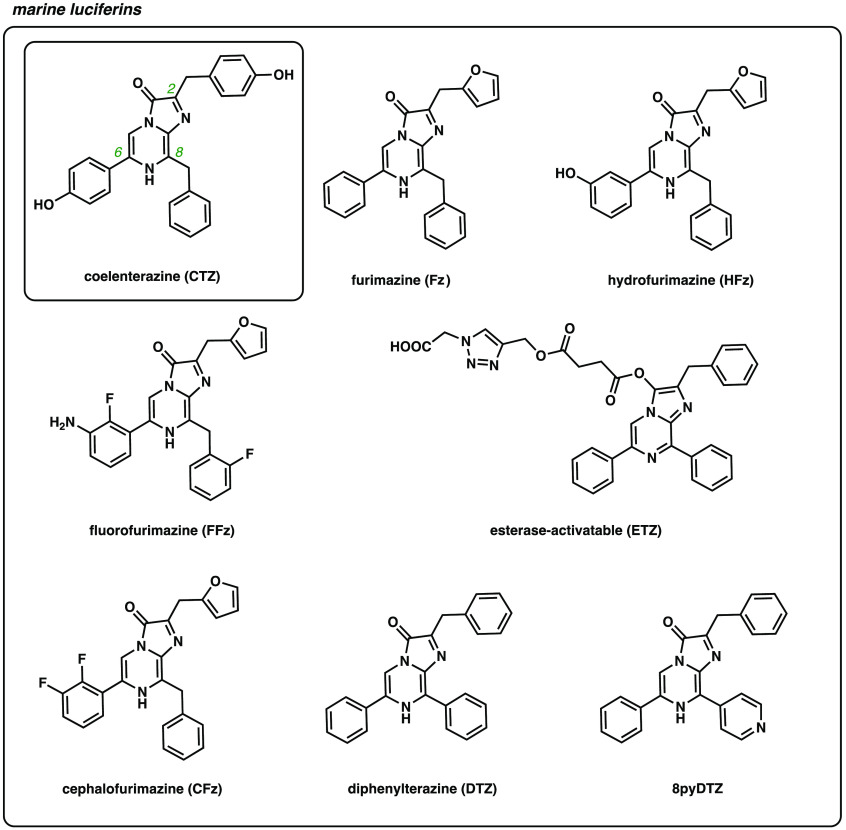
Luciferin analogs based on the native marine substrate, coelenterazine.

Faster-acting (and thus brighter) luciferases from marine organisms have also attracted attention for imaging *in vivo*. Included in this group are luciferases derived from *Renilla reniformis* (Rluc), *Gaussia princeps* (Gluc), and *Oplophorus gracilirostris* (OLuc). These enzymes oxidize a unique luciferin [coelenterazine (CTZ)] to produce blue light (450 to 500 nm, Fig. 3).^19^^–^^21^ No cofactors besides oxygen are required. The marine luciferases and CTZ have been similarly coopted for use in preclinical imaging, but early applications were more limited in scope.^8^^,^^22^^–^^24^ The probes are bright but give off primarily blue light, which is less tissue-penetrant. CTZ itself also suffers from poorer overall stability, solubility, and blood-brain-barrier penetration.^10^^,^^22^ In many cases, the luciferin must be delivered via i.v. or intracranial injection, which can hinder serial imaging experiments. Substrates with improved pharmacokinetic properties have thus long-been sought for neuroscience applications.

Multicomponent imaging has been another long standing challenge in BLI. Examining the intricate networks underlying learning, memory, and other functions requires simultaneous tracking of multiple cell types and biomolecular features. Since bioluminescent pairs emit broad emission spectra, they are often difficult to distinguish by color alone in deep tissue.^14^ Insect and marine luciferases can be readily resolved based on their different (i.e., orthogonal) substrates. There is no cross-reactivity between these two classes of reagents, enabling two cell types to be visualized simultaneously.^3^^,^^25^^,^^26^ Imaging beyond two targets, though, is far from routine.^25^ More orthogonal luciferases are thus required to examine brain circuits and other complex biology.

## 3 Improved Pharmacokinetics

### 3.1 Insect Luciferins

Recent efforts to enhance BLI sensitivity have focused on improving luciferin bioavailability, particularly in the brain (Fig. 2).^1^^,^^8^ One particularly effective strategy has involved replacing the 6′ hydroxyl group of D-luciferin with amino substituents. For example, Miller and coworkers generated a series of cyclic aminoluciferins with enhanced stability, permeability, and brain penetrance. One analog (CycLuc1) enabled imaging at a 20-fold lower dose than D-luciferin.^27^ Additionally, CycLuc1 provided more sensitive readouts in brain tissue, where D-luciferin has been historically limited. A related aminoluciferin (cybLuc) reported by Wu et al. showcased 20-fold brighter emission than D-luciferin at 0.01% of the standard dose.^28^ CybLuc further provided more signal *in vivo* (seven-fold higher signal compared to D-luciferin, even when used at a 10-fold lower dose).

Masking the charged carboxylate on D-luciferin has also been an effective strategy to improve luciferin bioavailability. Ester- and amide-based cages have both been used to increase cell permeability. Once inside the cell, the cages can be removed by cellular machinery (e.g., enzymes) to release active substrate. For example, the caged-ester analog, DMNPE-D-luciferin, 1-(4,5-dimethoxy-2-nitrophenyl)ester) can cross the cell membrane more efficiently than native D-lucifeirn.^29^ Once internalized, the luciferin ester is hydrolyzed by cellular esterases (or can be released by ultraviolet light) to supply active D-luciferin. DMNPE-D-luciferin has been employed for several neuronal imaging applications, including measuring ATP levels in neurons.^30^ Recently, Yadav et al. developed an isopropyl ester-caged analog that is more resistant to non-specific hydrolysis and can thus improve overall detection sensitivity.^31^ This cage is advantageous for a variety of activity-based sensing applications in neuroscience.

### 3.2 Marine Luciferins

CTZ, the substrate of marine luciferases, has similarly been tuned for improved pharmacokinetics. As noted earlier, this luciferin is more prone to autooxidation, leading to high background signal.^22^ In the past several years, improvements in analog stability and solubility, as well as enzyme turnover rates, have dramatically improved BLI (Fig. 3). In one notable example, Promega developed a novel CTZ analog, furimazine (Fz), which bears a furan group at the C-2 position. Modifications at this position are typically well-tolerated by luciferases, and also enhance luciferin stability.^32^ Fz exhibited increased stability and lower levels of autooxidation compared to CTZ. A complementary luciferase enzyme, NanoLuc, was further engineered to accommodate the modified luciferin scaffold.^33^ NanoLuc/Fz has gained wide-spread traction for imaging applications, given its overall brightness (∼150-fold brighter *in vitro* than Rluc/CTZ).

While enabling new areas of science, Fz is poorly soluble in aqueous solution, limiting the concentration that can be administered *in vivo*. Fz delivery to the brain and other tissues can be enhanced with common drug delivery formulations (e.g., polyethylene glycol-300 and poloxamer-407). Poloxamer-407 can further enable the sustained-release of luciferin, a beneficial feature for monitoring physiological events over long periods of time.^34^^–^^36^ Additional modifications to the luciferin core have also been investigated to improve substrate bioavailability. In recent work, two Fz analogs, hydrofurimazine (HFz) and fluorofurimazine (FFz), exhibited improved solubility and could be administered at higher doses *in vivo*.^35^ Interestingly, the added solubility did not translate to higher photon outputs in the brain. Both HFz and FFz provided brighter emission than Fz in the periphery, but reduced photon outputs and penetration were observed in the brain. These results highlight the importance of continued analog development and empirical testing of substrates for *in vivo* use.^34^

Similar to D-luciferin, marine luciferin analogs have been outfitted with various caging groups to improve stability and tissue targeting. Since the C3 imidazopyrazinone carbonyl is required for the light-emitting reaction, cages at this position can block pre-mature oxidation. For example, Tian et al. developed the analog ETZ, with an extended carboxylate at the C3 position to prevent luciferase-mediated or auto-oxidation. Upon *in vivo* administration, nonspecific esterase activity hydrolyzes the ester, liberating an active substrate. ETZ was shown to be a superior luciferin for imaging in the brain, based on its brightness and signal durability.^37^

## 4 Red-shifted Imaging Probes

### 4.1 Synthetic Modifications

#### 4.1.1 Insect luciferins

Neuroscience studies have benefitted from efforts to engineer luciferins that emit near-infrared (NIR, >650  nm) light. As noted earlier, such wavelengths can more effectively penetrate tissue to boost imaging sensitivity in the brain and other deep tissues.^14^ Improved red-emitting probes have resulted from a combination of both synthetic luciferin engineering and luciferase evolution. This section will primarily focus on efforts to chemically tune the luciferin: extending pi-conjugation, replacing electron donating groups, and modifying the core heterocycles.

Altering the luciferin chromophore is one common approach to achieve desirable spectral outputs (Fig. 2). This strategy was successfully used to generate the NIR analog AkaLumine-hydrochloride (AkaLumine-HCl). This luciferin has been widely used for a variety of sensitive *in vivo* studies.^38^ To generate AkaLumine-HCl, Kuchimaru et al. replaced the benzothiazole group of native D-luciferin with a dimethylaniline unit. Two vinyl units were used to link the aniline motifs to the luciferin thiazole. When incubated with Fluc, AkaLumine-HCl produced a substantial number of red photons ($\lambda_{\max}=677\;\mathrm{nm}$). The photon output was dimmer than Fluc/D-luciferin, but more sensitive imaging was possible in deep locales due the larger number of tissue-penetrant photons produced.^38^ Brightness was recouped via an extensive enzyme engineering campaign. The resulting luciferase, Akaluc, comprised 28 mutations.^39^ Akaluc/AkaLumine (AkaBLI) produced up to 1000-fold brighter emission *in vivo* than Fluc/D-luciferin and has been used for real-time imaging of brain cells in marmosets—an impressive feat for bioluminescent probes. AkaLumine-HCl has further been leveraged to visualize changes in BDNF expression in a living mouse brain, illuminating the role of this protein in neurological disorders.^40^

While AkaBLI is enhancing *in vivo* imaging studies, some limitations have hindered its widespread use. These include poor luciferin solubility, high hepatic signal, and some reported toxicities.^8^^,^^35^^,^^41^^–^^43^ A new luciferin analog, seMpai, was developed to mitigate background signal from the liver.^42^ This analog is structurally similar to AkaLumine, but features a pyridyl aniline moiety to increase hydrophilicity and reduce accumulation in hepatic tissue. SeMpai/Akaluc was capable of detecting micro-metastases *in vivo*, with no background signal or adverse effects observed in the mice.^42^

Additional modifications to the AkaLumine core have provided analogs with even further red-shifted emission. For example, Ikeda et al. fused various cyclic amino groups to AkaLumine to provide the NIRLuc series.^44^ These analogs exhibit NIR emission ($\lambda_{\max}=690\;\mathrm{nm}$) with native Fluc. When applied *in vivo* to image subcutaneous tumors, one NIRLuc analog displayed seven-fold brighter emission than D-luciferin, and emission on par with AkaLumine. The NIRLuc analog also showcased improved blood retention and higher Fluc affinity, resulting in more sustained bioluminescence at lower doses. In addition to cyclic amino modifications, Yadav et al. demonstrated that even a minor switch from an *N*,*N*-dimethyl to an *N*,*N*-diethyl moiety could shift the emission of AkaLumine by ∼10  nm.^45^ Further red-shifts have been achieved with added vinyl units, although these probes remain quite dim.^46^

Extending the pi-conjugation between the benzothiazole and thiazole motifs of D-luciferin itself has also provided probes with enhanced red emission. This was observed for infra-luciferin, an analog developed by Jathoul et al.^47^ Infra-luciferin displays an emission maximum of 706 nm when paired with a variant of Fluc, and this combination has been used for sensitive imaging in mouse models. Notably, this analog can provide different colors of light when paired with distinct luciferase enzymes.^48^ Such spectral differences are advantageous for visualizing multiple cell populations in the same mouse. Toward this end, the authors showcased dual color imaging in mice via infa-luciferin application.^49^

Other modified analogs of D-luciferin have similarly provided red-shifted emission. Hall et al. fused additional phenyl rings to the benzothiazole core to generate naphthyl-luciferins.^50^ Two of these analogs (${\mathrm{NH}}_{2}\text{-}\mathrm{NpLH}2$, OH-NpLH2) produced red-emission ($\lambda_{\max}=730\;\mathrm{nm}$, 743 nm) with a mutant click beetle luciferase, CBR2. These pairs represent among the most red-shifted luciferin/luciferase probes to date. Both were capable of imaging through black fur mice, and in the brain, albeit with dimmer photon outputs than Fluc/D-luciferin and Akaluc/AkaLumine. The attenuated emission *in vivo* could potentially be addressed with additional efforts to tune the scaffolds for improved bioavailability.

We and others have further been inspired to design new classes of red-emitting luciferins based on related fluorescent scaffolds. In particular, we were attracted to red-shifted coumarin analogs reported by the Schnermann group.^51^^,^^52^ These fluorophores are compact and comprise LUMO-lowering elements to red-shift emission. The coumarins were appended to the luciferin thiazoline via an intervening pi-wire, providing coumarin-luciferins (CouLucs). The CouLucs were accessed from short and scalable routes, and the pi-conjugation was readily extended. Each vinyl unit in the pi-wire evoked a spectral shift of ∼100  nm, similar to the trends seen with both AkaLumine and infra-luciferin analogs. CouLucs displayed emission ranging from 597 to 730 nm with various engineered Fluc enzymes, and were capable of imaging subcutaneous tumors in black fur mice with slightly stronger signal than Fluc/D-luciferin.^41^ Additionally, the distinct architectures of the CouLucs facilitated easy multiplexing with existing bioluminescent probes.

#### 4.1.2 Marine luciferins

Similar synthetic strategies have been employed to tune the emission properties of marine luciferin analogs (Fig. 3). Alterations at C8 have been commonly used to red-shift emission by ∼100  nm while maintaining reasonable light output.^53^^,^^54^ Along these lines, Yeh et al. installed a phenyl group at C8, extending the pi-conjugation of the luciferin, to generate the red-shifted analog diphenylterazine (DTZ).^55^ A complementary enzyme, teLuc, was engineered from NanoLuc to accommodate the altered scaffold. DTZ/teLuc exhibited light output with maximal emission at 502 nm. Impressively, this probe set exhibited 54-fold brighter emission than Fluc/D-luciferin in mouse tumor models. DTZ is poorly soluble compared to related luciferins, but Yeh et al. recently reported an improved variant comprising a C8 pyridyl motif (8pyDTZ). Pyridine groups readily form pyridinium salts at physiological pH, enhancing water solubility. 8pyDTZ was seven-fold more soluble than DTZ, and exhibited red-shifted emission ($\lambda_{\max}=520\;\mathrm{nm}$) with teLuc. However, the pyridyl modification perturbed enzyme turnover, resulting in attenuated photon output. Additional enzyme engineering was used to overcome this limitation, with the optimal enzyme termed LumiLuc.

The need for an expanded set of luciferase-luciferin probes has spurred additional efforts to generate custom tools. A particularly stunning example showcased a luciferase designed from scratch. Yeh et al. used a deep-learning based approach to generate LuxSit, a luciferase with high selectivity for DTZ.^56^ The designer enzyme was small (13.9 kDa) and thermostable, with a catalytic efficiency on par with native luciferases. Notably, this enzyme did not originate from a functionally annotated luciferase, but rather from a nuclear transport factor 2 (NFT2)-like sequence, broadening the suite of enzymes from which useful imaging probes might be found. *De novo* designed probes could dramatically expand the collection of tools available for neurobiology studies.

### 4.2 BRET Probes

**Fig. 4:**
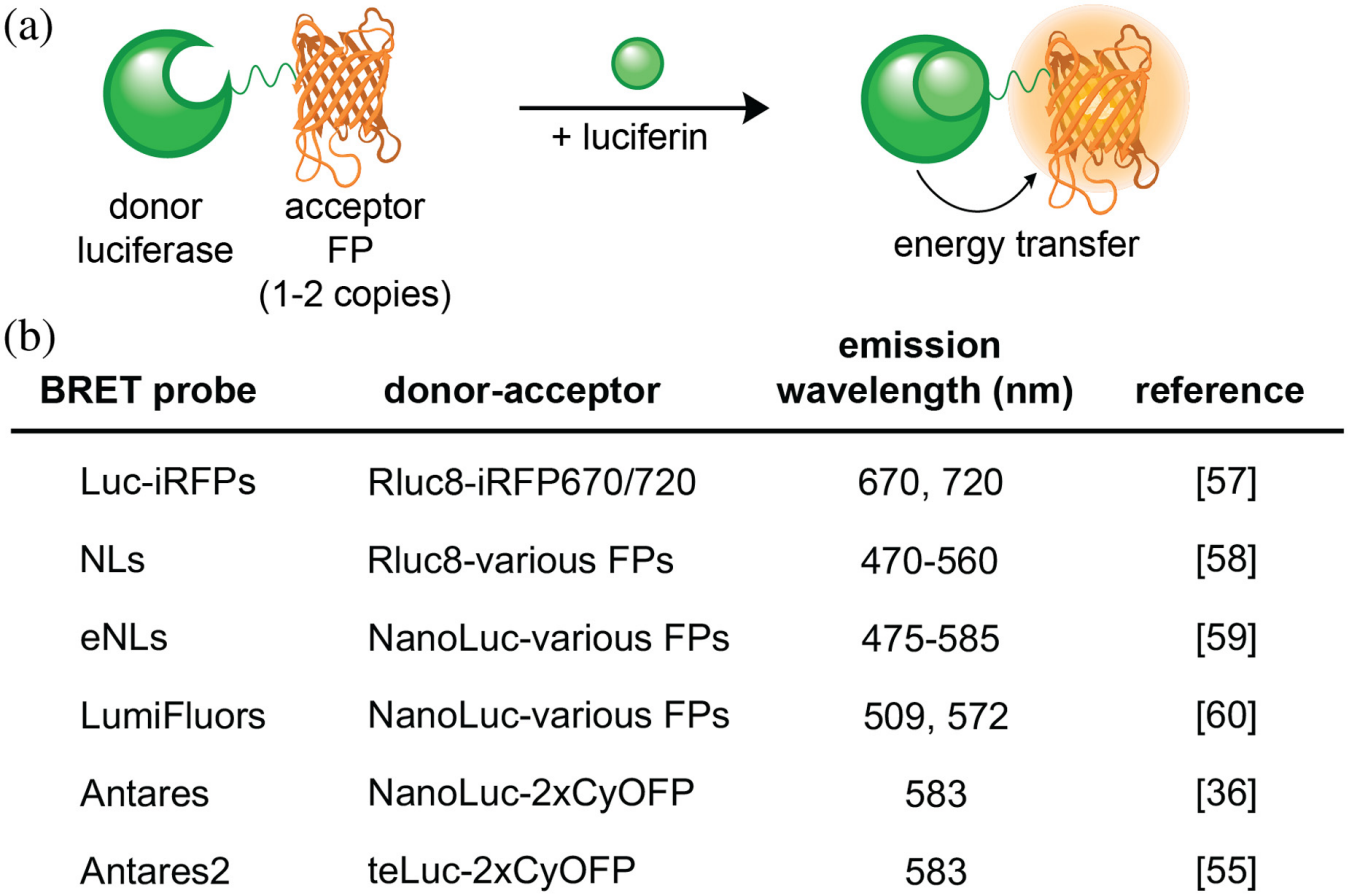
BRET-probe mechanism and properties. (a) Schematic of BRET-based probe design. A donor luciferase is fused with a FP acceptor. Upon luciferin administration, energy from the bioluminescent reaction is transferred to the FP. (b) Table of BRET probes with potential for brain imaging, with their donor-acceptor pairs and emission wavelengths noted.

In addition to luciferin modifications, spectral shifts can be achieved via bioluminescence resonance energy transfer (BRET). BRET couples luciferases as energy donors with fluorescent probes as acceptors, resulting in emission at longer wavelengths [Fig. 4(a)]. Several classes of BRET probes have been produced, with the majority comprising fluorescent proteins (FPs) as acceptors [Fig. 4(b)]. The efficiency of energy transfer is dependent on both the intermolecular distance and the spectral overlap of the donor and acceptor.

BRET has proven to be an effective strategy for red-shifting many bioluminescent probes, enhancing their utility *in vivo*.^10^ Particularly striking examples feature fusions of NIR fluorescent proteins to Renilla luciferase (Rluc). Rumyantsev et al. generated BRET constructs comprising iRFP670 or iRFP720 linked to a Renilla luciferase variant (RLuc8). The resulting BRET probe was used for sensitive imaging of tumor cells in mouse lungs.^57^ These probes could detect as few as $10^{4}$ cells in deep tissue, a 10-fold improvement in the number of cells that could be detected using the NIR fluorescent proteins alone. Several other fluorescent protein fusions to Rluc have also enabled bright and color-resolvable signals. These include the Nano-lantern series from Takai et al., which features yellow-green, cyan, and orange emitting Rluc-FP fusions. The Nano-lanterns exhibited 20-fold brighter emission than Rluc alone, and enabled multicolor imaging in live cells.^58^

Suzuki et al. expanded upon the Nano-lanterns to develop a series of NanoLuc-based BRET probes (enhanced Nano-lanterns, eNLs). The eNLs comprise five different fluorescent protein fusions and span an emission window of 475 to 585 nm.^59^ Related BRET probes (LumiFluors) have been reported by Schaub et al.^60^ LumiFluor variants (eGFP-NanoLuc, LSSmOrange-NanoLuc) have been successfully applied to visualize tumor cells in mice. These probes and the eNLs could be particularly useful for multiplexed imaging in the brain and other deep tissues, given recent advances in detection schemes (see below). BRET-based readouts from NanoLuc and a variety of FPs have also been exploited for numerous sensing applications (also discussed below).^10^^,^^61^

Chu et al. observed enhanced BRET efficiencies when NanoLuc was fused to more than one copy of the fluorescent protein acceptor. In this example, two copies of an optimized orange fluorescent protein, CyOFP1, were appended to the termini of NanoLuc.^36^ The resulting construct, Antares, exhibited superior sensitivity in deep tissue imaging experiments compared to classic *in vivo* probes (Fluc, among others). A similar engineering strategy was used with DTZ/teLuc to generate Antares2.^55^

**Fig. 5:**
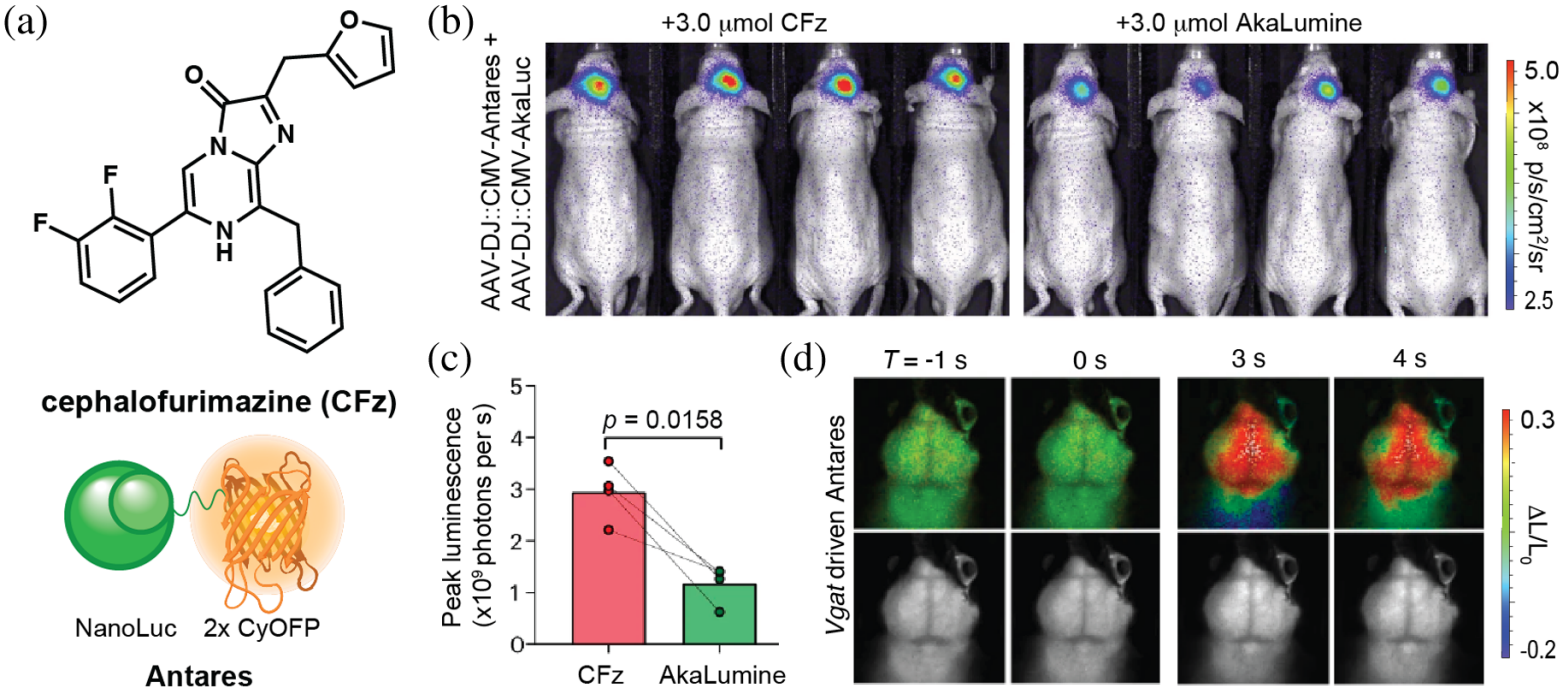
An optimized luciferin substrate for brain imaging. (a) Chemical structure of cephalofurimazine (CFz) and cartoon depiction of the corresponding BRET construct, Antares. (b) Representative bioluminescence images of Antares/CFz and Akaluc/AkaLumine in the hippocampus of mice. (c) Quantification and comparison of peak signal intensities. (d) Stimulation-dependent brain bioluminescence responses of *Vgat*-Antares transgenic mice at various time points. (b)–(d) Adapted with permission from Su et al.^34^ Published by Nature Publishing Group under a Creative Commons Attribution 4.0 International License.

Further improvements in imaging brain targets are expected from the merger of optimized BRET constructs with tissue-penetrant luciferins. Su et al. recently screened a panel of luciferin analogs to identify substrates for Antares that could provide enhanced imaging in the brain. One of the hits was a difluorinated substrate Fz analog, cephalofurimiazine (CFz) [Figs. 5(a) and 5(b)]. Antares/CFz achieved 20-fold greater signal output in the brain compared to Fluc/D-luciferin and enabled video rate monitoring of cells.^34^ Antares/CFz matched Akaluc/AkaLumine in terms of overall sensitivity in the brain, despite its more blue-shifted emission. The two probe sets are also orthogonal to one another, providing an avenue for dual imaging [Figs. 5(b) and 5(c)]. The extraordinary brightness of Antares/CFz can also be leveraged for sensitive tracking of gene expression. Along these lines, Su et al. employed Antares/CFz to detect neuronal activity via stimulation-dependent vesicular GABA transporter (*Vgat*) expression. In these studies, bioluminescent light output was used to track sensory stimulation in the brains of mice [Fig. 5(d)].

CFz has enabled other imaging studies in the brain. Recently, Wu et al. paired CFz with NanoLuc to generate a kinase-sensing probe.^62^ This probe features a phosphopeptide-binding domain (PBD) inserted between two fragments of NanoLuc. Binding of the PBD to phosphorylated substrates prevents NanoLuc complementation when kinase activity is high. When kinase activity is decreased, the dephosphorylation event triggers a conformation shift to favor NanoLuc complementation, and hence light production. These probes, kinase-modulated bioluminescent indicators (KiMBIs), were used to monitor drug activity in the brain of living mice. The KiMBis successfully identified temuterkib as a promising brain-active ERK inhibitor.

## 5 Examples of Functional Bioluminescent Probes for Brain Imaging

### 5.1 Calcium Sensors

Noninvasive BLI is advantageous for imaging metabolites and signaling molecules relevant to neurobiology. Calcium (${\mathrm{Ca}}^{2+}$) is one prominent example involved in a variety of neuronal processes, including synaptic activity and memory formation.^63^ ${\mathrm{Ca}}^{2+}$ has long been a target for BLI sensor development, with common designs based on naturally occurring calcium binding domains (e.g., calmodulin/M13 peptide). Conformational shifts in these sensors drive luciferase fragment complementation or changes in fluorescent protein proximity, resulting in altered optical readouts.^59^^,^^64^^–^^68^ One example features the enhanced Nano-lantern GeNL, with calmodulin and M13 inserted into the NanoLuc fusion.^59^ This probe exhibits a large change in signal output (500%) in the presence of calcium, enabling dynamic tracing in neurons. Oh et al. installed the same calcium-sensing motif into Antares, yielding an orange calcium-modulated bioluminescent indicator (OrangeCaMBI). OrangeCaMBI exhibits an approximate 8-fold change in photon production upon calcium binding.^69^ A slightly modified version of this sensor, OrangeCaMBI110, was paired with the optimized NanoLuc substrate, CFz, to measure sensory-evoked neuronal activity in the mouse brain.^34^

Both tunable and red-shifted emissions are important features for multiplexed sensing and brain imaging. Methods to easily tune emission color without extensive protein engineering are particularly valuable. Toward this end, Hiblot et al. developed a NanoLuc-HaloTag fusion, H-Luc, that can be coupled with a variety of synthetic fluorophores to provide a range of spectral outputs.^70^ Mertes et al. further combined H-Luc with calcium-sensing indicators (MaPCa),^68^ providing a palette of sensors for noninvasive detection. It should also be noted that BRET-based sensors exist for other neuro-relevant metabolites and activities, beyond those highlighted here.^10^ One notable example is a bioluminescent voltage indicator that can record brain activity in mobile mice.^61^

### 5.2 Optogenetic Probes

Designer luciferins are useful not just for imaging in the brain, but also for driving biological processes. Inspiration has come from the field of optogenetics, where photons are used to activate photoswitches and other light-responsive elements. Conventional optogenetic probes rely on external photon delivery via implanted fiber optic devices or LEDs. Bioluminescent optogenetic (BL-OG) probes offer some advantages in this context since no invasive light delivery is required. Rather, simple application of a luciferin provides the necessary energy.

BL-OG probes have been employed to control neuron behavior. Early work featured luciferase (Gluc) fusions to opsins, generating luminescent opsins (LMOs).^71^^–^^73^ These LMOs were capable of both neuron activation and silencing *in vitro* and *in vivo*. More recent work has featured combinations of engineered Gluc variants with modified ChRs for improved function, including pro-longed neuromodulation.^72^^,^^74^

The ability to manipulate specific neuronal cell populations renders bioluminescent optogenetics a promising tactic for treating neurological disorders. Recently, Peterson et al. used LMOs to activate neuron populations in rats post spinal cord injury.^75^ Rats receiving the neural stimulation showed improved locomotor recovery, highlighting the potential for bioluminescent optogenetics to be used for spinal injury rehabilitation. Challenges still remain, though, regarding the spatial and temporal activation of LMOs and related BL-OG tools. Without additional control over luciferin tuning and release, it is difficult to precisely manipulate defined populations of cells or biomolecules.

Flavin-binding proteins, such as the LOV domain, have also garnered attention as BL-OG tools.^76^^,^^77^ LOV domains are involved in circadian rhythms and stress responses, and are found in many different organisms.^78^^–^^80^ These proteins undergo a key cysteine addition upon flavin excitation, triggering a conformational change that initiates the phototransduction cascade.^81^^,^^82^ LOV domains have recently been coopted for driving cellular networks via bioluminescent stimulation.^83^^,^^84^ A particularly noteworthy example is SPARK, a protein-protein interaction reporter that requires blue light input. SPARK features a transcription factor bound to the membrane via the β-adrenergic receptor. This GPCR is further tethered to a LOV domain harboring a TEV protease-cleavage site. Upon blue-light activation, the conformational switch of the LOV domain reveals the cleavage site, resulting in release of the transcription factor.^83^^,^^84^ Kim et al. showed that bioluminescence (via a luciferase fusion) can drive the conformational switch.^83^^,^^84^ Addition of the luciferin (and ensuing BRET) activated the LOV domain and ultimately resulted in downstream transcription. This bioluminescent platform was applied to high-throughput screening for GPCR antagonists and detection of trans-cellular contacts. A bioluminescent-responsive LOV domain has also been developed for optogenetic control of proximity labeling.^85^

### 5.3 Caged Luciferin Probes

Caged luciferin molecules represent another toolset to study dynamic neurological processes. Such probes have been applied to monitoring and tracking enzyme activity, metabolites, and other biomolecules *in vivo*. The same modifications that have been used to enhance stability (see Sec. 3) can also be triggered to release active luciferin in response to cellular species. This strategy most commonly involves caging the 6′-hydroxyl or carboxylic acid moieties of D-luciferin, as such modifications often inhibit substrate processing by luciferase, and hence diminish bioluminescence.^11^^,^^86^ In the presence of a specific enzyme or biomolecule, though, the cages are released to reveal viable luciferins. These molecules are then used by luciferases to emit light. The intensity of light output correlates to the amount of free luciferin, thus reporting on the amount of enzyme activity or target biomolecule.

#### 5.3.1 Enzyme detection

**Fig. 6:**
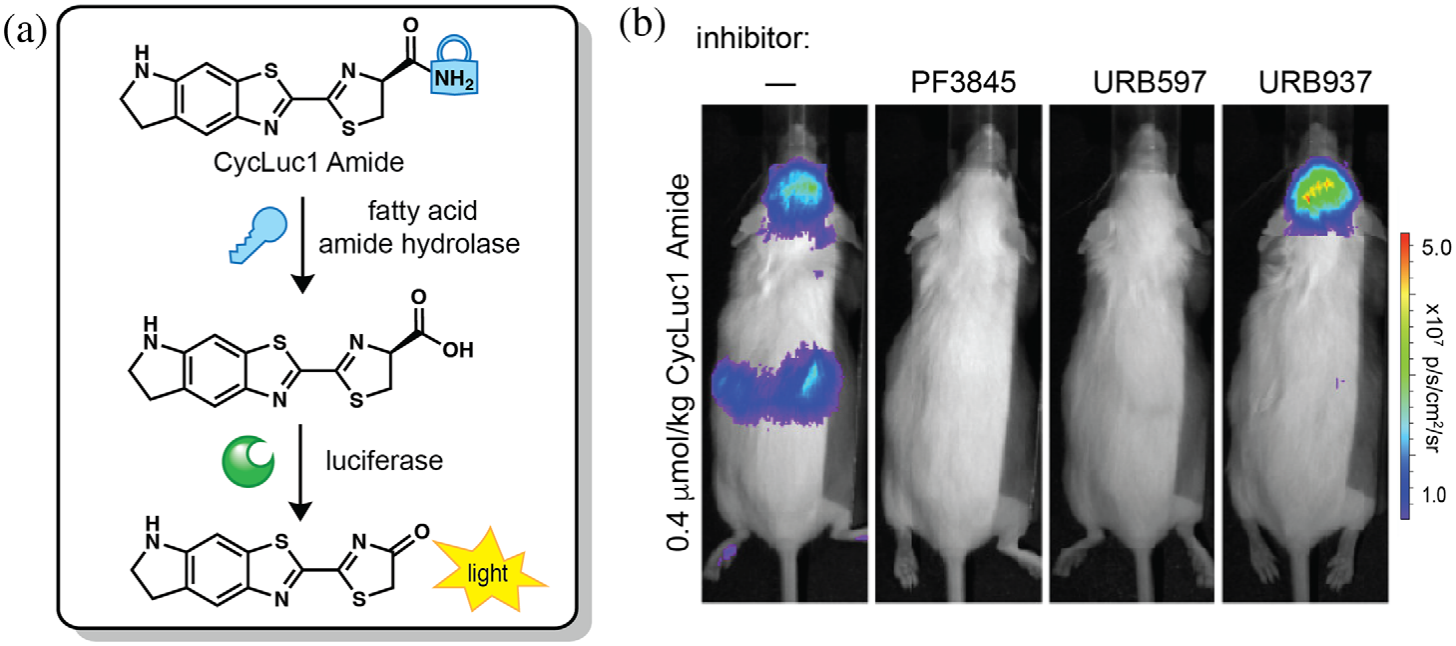
Caged luciferins to detect enzyme activity in the brain. a) Schematic of enzyme uncaging. CycLuc1 is caged with an amide (CycLuc1 Amide) that can be cleaved by fatty acid amide hydrolase (FAAH). Upon enzyme cleavage, active CycLuc1 is released and can produce light with luciferase. b) BLI with CycLuc1 Amide in luciferase-expressing mice treated with: no inhibitor (vehicle only), FAAH inhibitors PF3845 or URB597, or brain-impermeable FAAH inhibitor URB937. Adapted with permission from Mofford et al.^88^ Copyright Journal of American Chemical Society.

Caged luciferins are valuable probes for measuring enzymatic activity in biological environments. Enzyme-activable luciferins are typically crafted by caging the 6′-hydroxyl or carboxylic acid group of D-luciferin with an enzyme-labile functional group or substrate. In the presence of the enzyme of interest, the cage is removed to reveal active luciferin. One example with relevance to neurobiology involves fatty acid amide hydrolase (FAAH). FAAH inhibition is of therapeutic interest to treat pain, anxiety, and cannabinoid dependence.^87^ Toward this end, Mofford et al. developed amide-caged luciferins to detect FAAH activity [Fig. 6(a)].^88^^,^^89^ The luciferin amides were hydrolyzed by FAAH, becoming active Fluc substrates. The luciferins thus enabled detection of FAAH activity in live mice, achieving sensitive imaging in the brain and providing a platform for drug screening [Fig. 6(b)].

Similar caged probes have been developed to detect proteases with established roles in neurodegenerative disease.^90^ For example, furin and various caspases have been detected using aminoluciferins outfitted with appropriate peptide sequences (e.g., Z-DEVD or acetyl-RVRR/RYKR).^91^ These probes were successfully deployed in oncology models, facilitating the discovery of new drugs. Peptide-caged luciferins are similarly poised to examine neurodegenerative disease, as proteolysis mechanisms underlie the accumulation of toxic peptides and other aggregates.

#### 5.3.2 Analyte detection

In addition to studying enzyme activity, caged bioluminescent probes have also found broad application in monitoring biomolecule activity. Zuo et al. reported the first caged bioluminescent probe for studying norepinephrine (NE)—a critical neurotransmitter in the brain.^92^ Norepinephrine levels have also been associated with a multitude of neurodegenerative disease and mental illness. To detect this biomolecule, the authors developed an NE-responsive caged luciferin. The 6′-hydroxyl group of D-luciferin was masked with a p-toluenethiol moiety; this group can be removed via NE-triggered C-S bond cleavage. The caged luciferin was successful in monitoring NE in living mice, including the brain. A similar strategy could be used to report on other critical neurotransmitters, such as dopamine, GABA, acetylcholine, and more.

Additionally, luciferins have been developed to detect a wide variety of metals. Copper and iron, in particular, play significant physiological roles in brain development and function.^93^ Several metals have further been implicated in neurodegenerative disease.^94^ Heffern et al. constructed a copper-caged luciferin, CCL-1, leveraging a Cu(I)-dependent oxidative cleavage reaction to release D-luciferin.^95^ Physiological changes in labile Cu(I) levels were detected in mice under both copper deficiency or overload. CCL-1 was also applied to monitor a diet-induced mouse model of nonalcoholic fatty acid liver disease. In this case, Aron et al. designed an iron bioluminescent probe that enabled longitudinal monitoring of labile iron in living animals.^96^ Their probe, ICL-1, featured an Fe^2+^-reactive endoperoxide trigger to release aminoluciferin. ICL-1 was also applied to study a model system of bacterial infection and iron accumulation in infected tissue.

## 6 Multicomponent Imaging

**Fig. 7:**
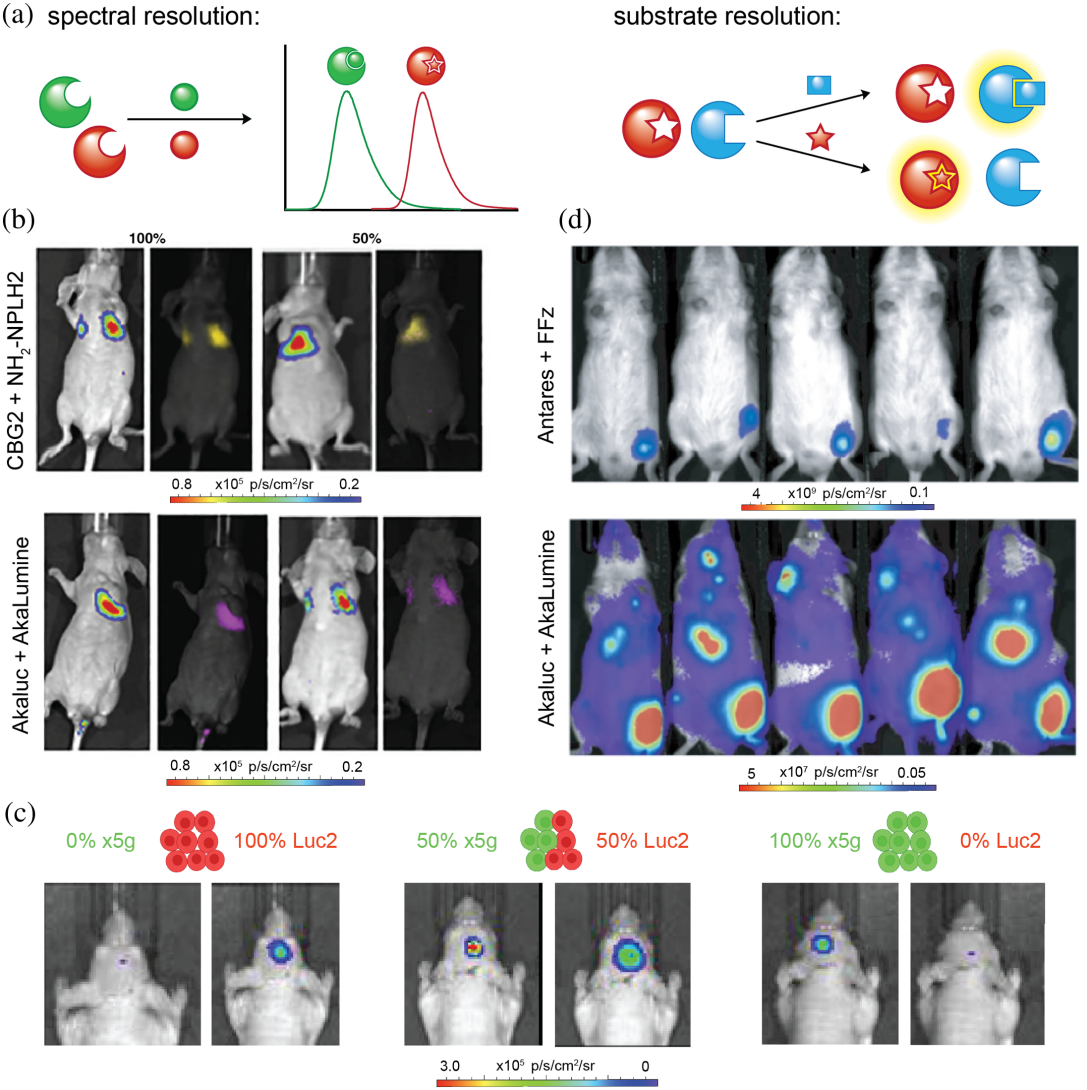
Multicomponent BLI strategies and applications. (a) Cartoon representations of spectral resolution and substrate resolution. (b) Spectral unmixing *in vivo* for cell populations comprising 100% (left) or 50% (right) mixtures of CBG2- or Akaluc-expressing HEK cells, treated with NH2NpLH2 or AkaLumine. Adapted with permission from Zambito et al.^99^ Published by Elsevier under a Creative Commons Attribution 4.0 International License. (c) Spectral unmixing of two NSC populations implanted in different ratios in the cortex of mice. Adapted with permission from Aswendt et al.^101^ Published by SPIE under a Creative Commons Attribution 4.0 International License. (d) BLI in mice engrafted with Antares-expressing tumors and injected with Akaluc-expressing CAR-T cells. Adapted with permission from Su et al.^35^ Copyright Springer Nature.

The rapidly growing palette of luciferin probes can enable multiplexed detection in the brain. Such studies have the potential to expand our understanding of complex neurochemical pathways and processes. One approach to multi-component imaging involves spectral resolution of tissue-penetrant bioluminescent probes [Fig. 7(a)]. Red-emitting tools can be used in tandem with more blue-shifted pairs, and the wavelengths can be distinguished using filter sets or spectral unmixing algorithms.^97^ One example from Moroz et al. demonstrated four-component imaging of distinct subcutaneous cell implants. Two marine-based probes (vargulin/Cypridina luciferase, CTZ/Rluc) and two insect-based probes (D-luciferin/CBR, D-luciferin/CBG) were used to identify the cell populations. Applications were limited to surface tissues, though, owing suboptimal photon outputs.^98^

Multi-component imaging has been more readily achieved in deep tissue with optimized red-shifted probes. Zambito et al. performed dual-BLI in lung tissue [Fig. 7(b)] using a custom spectral unmixing algorithm.^99^ Infra-luciferin has also been successfully employed for multicomponent imaging *in vivo*, as it can produce a range of colors when paired with various enzymes. Leveraging these unique spectral properties, Stowe et al. performed dual-BLI using infra-luciferin paired with green- and red-emitting Fluc mutants.^49^ Kleinovink et al. further demonstrated that CycLuc1 and D-luciferin could be employed for dual-BLI to visualize T cell localization and activation.^100^ Dual-color BLI in the brain was also recently achieved. Aswendt et al. used Fluc mutants engineered to emit different colors of light with D-luciferin. The probe sets successfully distinguished two neural stem cell populations in the mouse brain, when spectrally unmixed [Fig. 7(c)].^101^ Unmixing additional probe sets is possible, but has been difficult to achieve in practice owing to the broad, overlapping emission spectra of most bioluminescent tools.^14^

An alternative approach to multiplexed imaging involves substrate resolution. Luciferases that harbor selectivity for distinct luciferins (i.e., orthogonal pairs) can be discriminated based on the type of luciferin supplied [Fig. 7(a)]. This is most easily accomplished by pairing enzyme-substrate pairs from different families. As previously mentioned, insect and marine based probes exhibit near-perfect orthogonality. For example, in the first report of dual-BLI, Bhaumik and Gambhir used Fluc/D-luciferin and Rluc/CTZ to visualize subcutaneous implants in mice.^23^ Engineered bioluminescent probes have also been used for multicomponent imaging. Su and coworkers used Antares/fluorofurimazine (FFz) in combination with Akaluc/AkaLumine to image tumor size and CAR-T cells in the same mouse [Fig. 7(d)].^35^

One limitation for substrate-based resolution is the need to wait for substrate clearance before administration of the second analog, greatly lengthening imaging times to hours or days.^35^^,^^99^^,^^102^ Our lab has recently developed a substrate-unmixing algorithm that can recognize differences in light emission profiles to enable detection of multiple luciferases, without the need to wait for substrate clearance. Using this algorithm, imaging of up to four luciferases was achieved *in vitro*, and two luciferases *in vivo*, with imaging on the minutes-to-hours timescale.^102^ This rapid multicomponent platform can aid in visualizing short-term changes in cell growth or gene expression.

## 7 Conclusions and Future Directions

With its high sensitivity and noninvasive readouts, bioluminescence remains one of the go-to technologies for imaging in whole animals. Applications in the brain have been slower to develop, though, due to the suboptimal emission profiles, pharmacokinetics, and multiplexing capabilities of luciferin analogs. Recent advances in probe design have begun to address these limitations, increasing the scope of BLI. The past few years alone have provided an extensive palette of new luciferins for multi-spectral detection, improved brain penetrance, and analyte sensing. This expanding toolkit will enable the visualization of new, complex cellular interactions in the brain and other deep tissues.

As BLI applications in neuroscience grow, more extensive efforts to optimize probes are needed. New luciferin analogs would benefit from radioactive tracing studies to assess tissue biodistribution in living animals. Standard formulations could also improve the delivery and bioavailability of new luciferins. Drawing inspiration from drug delivery vehicles, the formulations could further enable more targeted release and tunable kinetics. Additionally, more information on the acute and long term toxicity profiles of various analogs would aid future molecular design efforts.

Future studies will also benefit from ongoing efforts to build custom luciferase and luciferin architectures. More robust and red-emitting probes will provide added sensitivity for imaging key metabolites and signaling molecules. Additional analyte- and enzyme-responsive molecules will enable biochemical processes to be studied in real time. Designer luciferase-luciferin pairs will further enable multiplexed detection of many neurobiological networks. With this growing toolkit, bioluminescence is poised to deliver additional new insights into neuroscience.

## Data Availability

No new data are reported in this paper.
